# Transcriptome profiling and gene expression analyses of eggplant (*Solanum melongena* L.) under heat stress

**DOI:** 10.1371/journal.pone.0236980

**Published:** 2020-08-11

**Authors:** Aidong Zhang, Zongwen Zhu, Jing Shang, Shengmei Zhang, Haibin Shen, Xuexia Wu, Dingshi Zha

**Affiliations:** 1 Horticultural Research Institute, Shanghai Key Laboratory of Protected Horticultural Technology, Shanghai Academy of Agricultural Sciences, Shanghai, China; 2 College of Fisheries and Life Science, Shanghai Ocean University, Shanghai, China; 3 College of Horticulture, Nanjing Agricultural University, Nanjing, China; Texas Tech University, UNITED STATES

## Abstract

Global warming induces heat stress in eggplant, seriously affecting its quality and yield. The response to heat stress is a complex regulatory process; however, the exact mechanism in eggplant is unknown. We analyzed the transcriptome of eggplant under different high-temperature treatments using RNA-Seq technology. Three libraries treated at high temperatures were generated and sequenced. There were 40,733,667, 40,833,852, and 40,301,285 clean reads with 83.98%, 79.69%, and 84.42% of sequences mapped to the eggplant reference genome in groups exposed to 28°C (CK), 38°C (T38), and 43°C (T43), respectively. There were 3,067 and 1,456 DEGs in T38 vs CK and T43 vs CK groups, respectively. In these two DEG groups, 315 and 342 genes were up- and down-regulated, respectively, in common. Differential expression patterns of DEGs in antioxidant enzyme systems, detoxication, phytohormones, and transcription factors under heat stress were investigated. We screened heat stress-related genes for further validation by qRT-PCR. Regulation mechanisms may differ under different temperature treatments, in which heat shock proteins and heat stress transcription factors play vital roles. These results provide insight into the molecular mechanisms of the heat stress response in eggplant and may be useful in crop breeding.

## Introduction

Plants grow in an open environment that contains various abiotic and biotic stresses [1]. These stresses adversely affect plants and promote the evolution of defense mechanisms to cope with stress. Eggplant (*Solanum melongena* L.), a thermophilic plant, is an annual herb in the Solanaceae family. With increased global warming, the climate is gradually becoming warmer. Temperatures of 38°C and even as high as 43°C in the summer can seriously inhibit growth of seedling, flower development, and eventually impact the quality and yield of eggplant [2, 3].

Heat stress can affect the morphological and physiological characteristics of plants, including their external morphology, cell membrane system, antioxidant system, and osmotic adjustment. The response to heat stress is a complex regulatory process involving multiple signal transduction and genes [4]. There are known four pathways, including heat shock transcription factor-heat shock protein (Hsf-Hsp) pathway, Calcium ion-calmodulin (Ca^2+^-CaM) pathway, reactive oxygen species pathway, and hormone pathway, to regulate heat stress [5, 6]. Under heat stress, calcium channels on the cytoplasmic membrane open, leading to calcium influx and active calcium-dependent protein kinase and calcium/calmodulin-binding protein kinase and eventually initiate the expression of downstream heat stress response genes [7]. Plant accumulate proline, trehalose, solute protein, organic acids, and ployols, which mediate osmotic potential, protect membrane and alleviate osmotic stress, suffering from heat stress [8, 9]. Reactive oxygen species scavenging enzymes, such as superoxide dismutase (SOD), peroxidase (POD), ascorbate peroxidase (APX), glutathione reductase (GR), monodehydroascorbate reductase (MDAR), and glutathione peroxidase (GPX), are also known to be heat stress inducible factors [10]. Heat shock signals are transmitted to the nucleus and combined with heat shock-related genes to regulate their expression levels [11]. Plant hormones, such as abscisic acid, salicylic acid, and ethylene, also play important roles in heat shock signal transduction [12]. Hsfs, the terminal components of the heat shock signaling pathway, can directly regulate the expression levels of heat shock response genes [13]. Hsps are the most important heat stress proteins regulated by Hsfs and protect cells against the negative effects of high temperature [14]. Therefore, the Hsf-Hsp pathway is the main pathway responding to heat stress and plays a major role in the heat tolerance of plants.

Transcriptomics is a discipline that studies the transcription and regulation of genes and is important in functional genomics analyses [15]. Next-generation high-throughput sequencing technology, referred to as RNA-Seq, has been used to investigate gene expression in many plants, such as grape (*Vitis vinifera*), arabidopsis (*Arabidopsis thaliana*), cucumber (*Cucumis sativus*), pepper (*Capsicum annuum*), and cotton (*Gossypium hirsutum*) [16–20]. The complete genome of eggplant has been sequenced [21], making it more convenient to perform transcriptome analyses of eggplant.

Heat stress is an abiotic stress that is widely studied in plant. Recently, transcriptome analysis about Chieh-Qua (*Benincasa hispida* Cogn. var. Chieh-Qua How) displayed differentially expressed genes (DEGs) related to Hsps, ubiquitin-protein ligase, transcription factors (TFs), and pentatricopeptide repeat-containing proteins were significantly changed after heat stress [22]. A comprehensive analysis of the Korean fir (*Abies koreana*) genes expressed under heat stress using transcriptome analysis revealed 204 transcription factors and 189 Hsps as differentially expressed [23]. Transcriptomic analysis of the maize (*Zea mays* L.) inbred line B73 found 167 putative TFs response to heat stress at the seedling stage [24]. Our previous research has illustrated expression of genes related to anthocyanin biosynthesis in eggplant fruit under high-temperature stress [25]. Although much effort has been put into elucidating the molecular mechanisms under heat stress in plant, gene expression at different development stages in eggplant is still not fully understood. In this study, we analyzed the transcriptome of eggplant seedlings with heat stress using RNA-Seq technology with the Illumina HiseqXTen platform (San Diego, CA, USA). We constructed and sequenced cDNA libraries from three samples treated at different temperatures. We identified thousands of DEGs, which were evaluated by Gene Ontology (GO) and Kyoto Encyclopedia of Genes and Genomes (KEGG) enrichment analyses. We further analyzed the expression pattern of genes related to transcription factors, antioxidant enzyme systems, detoxication, phytohormones, and heat stress. These results provide insight into the molecular mechanisms of the heat stress response in eggplant and may be useful in crop breeding.

## Materials & methods

### Plant materials and treatments

The eggplant variety “Tewangda”, which was obtained from the Horticultural Research Institute, Shanghai Academy of Agricultural Sciences (Shanghai, China), was used in this study. The plants were grown in an environmentally controlled growth chamber programmed for cycles of 16 h of light (28°C) and 8 h dark (25°C) with a relative humidity of 60–70%. When the seedlings had four true leaves, they were transferred to growth chambers at 38°C and 43°C respectively. Each sample with 10 seedlings was collected after 3 h of different temperature treatments for RNA extraction. 10 seedlings per sample grown in a 28°C chamber using as control were also collected for RNA extraction.

### RNA extraction

Total RNA was extracted with the mirVana miRNA Isolation Kit (Ambion, Austin, TX, USA) and purified with an RNeasy Plant Mini Kit (Qiagen, Hilden, Germany) according to the manufacturer’s instructions. Three independent experimental replicates were used for each temperature treatment and control. Total RNA was quantified with a NanoDrop spectrophotometer (Thermo Fisher Scientific, Waltham, MA, USA). The A260/280 ratios of samples were all above 2. The 28S/18S ratio and the RNA integrity number (RIN) values were determined using an Agilent 2100 system (Agilent, California, USA).

### Preparation of cDNA library and sequencing

cDNA sequencing libraries were constructed using a TruSeq Stranded mRNA LTSample Prep Kit (Illumina). In briefly, mRNA was purified from 4 μg total RNA using poly-T oligo-attached magnetic beads and then broken into short fragments. First-strand cDNA was synthesized with SuperScript II Reverse Transcriptase (Invitrogen, Carlsbad, CA, USA) using the short fragments as templates. Second-strand cDNA was synthesized using DNA polymerase I and RNase H. The double-strand cDNA was purified, poly (A) was added to the end, and then the fragments were connected with adapters [26]. The libraries were sequenced with an Illumina HiseqXTen after quality control analysis using an Agilent 2100 Bioanalyzer (Santa Clara, CA, USA). Sequencing generated 100-bp paired-end reads as raw reads.

### RNA-Seq data analyses and DEG identification

To further analyze the RNA-Seq data, clean reads were obtained by removing reads containing adapters or poly-N and low-quality reads from among the raw reads. Clean reads were mapped to eggplant genome sequences (http://eggplant.kazusa.or.jp/index.html) [21] by HISAT2 [27] software. String Tie [28] was used to reconstruct the transcripts, and Cuffcompare (Cufflinks tools) [29] was used to compare reconstructed transcripts to the reference annotation; next, we selected ‘u’, 'i', 'o', and 'j' class code types as novel transcripts (http://cole-trapnell-lab.github.io/cufflinks/cuffcompare/index.html). CPC [30] was used to predict the coding potential of the novel transcripts, and then we merged the coding novel transcripts with reference transcripts to obtain a complete reference. Downstream analysis was performed based on this reference.

The gene expression level was indicated by the FPKM (fragments per kilobase per million mapped fragments) value [31]. To calculate the different expression levels of genes among the samples, htseq-count [32] was used to acquire the number of reads in each sample. Two functions (estimatSizeFactors and nbinomTest) in the DESeq (2012) R package were used to normalize the data and calculate the *p*-value and fold-change. A *p*-value <0.05 and fold-change >2 or <0.5 were set as thresholds for significantly differential expression.

### Annotation and classification of DEGs

Sequences of the DEGs were aligned to the KEGG database by BLASTX, retrieving proteins with the highest sequence similarity with the given sequences along with functional annotations for their proteins [33]. The GO annotations of the DEGs were obtained using the Blast2GO program [33, 34]. Web Gene Ontology Annotation Plot software was used to perform GO functional classification with a Pearson Chi square test [33, 35]. The DEGs were mapped to GO terms according to the analyses, and the numbers of DEGs in each term were calculated [33].

### Validation of gene expression profile by qRT-PCR

qRT-PCR was performed to verify the accuracy of 22 genes’ expression profile obtained from the RNA-Seq data. Total RNA was extracted as described above. First-strand cDNA was synthesized from 2 μg of total RNA by M-MLV reverse transcriptase (Promega, Madison, WI, USA) and oligo (dT)18 primers in a 25-μL reaction system. Real-time PCR was performed with SYBR Green PCR mix (Takara, Shiga, Japan) according to Zhang et al. (2016) [36] using Roche LightCycler 480 (Roche, Basel, Switzerland). Three replicates per sample was used in qRT-PCR. *SmEF1a* (Sme2.5_01406.1_g00001.1) was used as an endogenous control gene for qRT-PCR analyses. Relative expression levels of the target genes were calculated by the 2-ΔΔCt method. The primers used are listed in S1 Table.

## Results

### Transcriptome analyses of eggplant under normal and heat stress conditions

To understand the molecular mechanisms of the response to heat stress in eggplant, cDNA libraries were generated from the leaves of eggplants grown at 38°C (T38), 43°C (T43), and 28°C (CK) for 3 h. The nine libraries were sequenced on the Illumina HiseqXTen platform. After removing the adapter sequences, poly-N and low-quality reads from the raw data, the quality of the clean data was assessed, with the results shown in Table 1. There were 40,733,667, 40,833,852, and 40,301,285 clean reads (SRA accession: PRJNA613773) containing 6,110,050,050; 6,125,077,800; and 6,045,192,750 clean bases in the CK, T38, and T43 groups, respectively. The Q20 of clean reads (the proportion of number of bases with a quality value greater than 20 compared to the total number of bases in the clean reads) in these data was more than 98%. The GC contents of the three samples were 43.78%, 42.95%, and 43.53%, respectively. As shown in Table 2, 83.98%, 79.69%, and 84.42% of the clean reads were mapped to the reference genome of eggplant, including 80.72%, 76.90%, and 81.40% uniquely mapped reads in three samples.

**Table 1:** Summary of transcriptome sequencing data.

sample name	clean reads	clean bases	Q20 (%)	GC (%)
CK	40733667	6110050050	98.18	43.78
T38	40833852	6125077800	98.23	42.95
T43	40301285	6045192750	98.21	43.53

**Table 2:** Total number and percentage of clean reads that were mapped to reference genome.

Map to genome	CK sample		T38 sample		T43 sample	
	Number of reads	percentage	Number of reads	percentage	Number of reads	percentage
Tatal clean reads	40733667	100%	40833852	100%	40301285	100%
Tatal mapped reads	34209931	83.98%	32538934	79.69%	34022177	84.42%
Tatal unmapped reads	6523736	16.02%	8294918	20.31%	6279108	15.58%
Uniquely mapped reads	32878359	80.72%	31402986	76.90%	32807106	81.40%

### Prediction of new transcripts

New transcripts are those that are not annotated in databases and may be unknown genes or new splicing subtypes of known genes. After mapping to the reference genome, we reconstructed transcripts of the three samples. The reconstructed transcripts were compared to reference annotation information and the coding potential was predicted with CPC software [30]. Eventually, we obtained 25,482 new transcripts containing 20,641 predicted coding transcripts and 4,841 non-coding transcripts (S2 Table).

### Differential expression analyses

**Fig 1:**
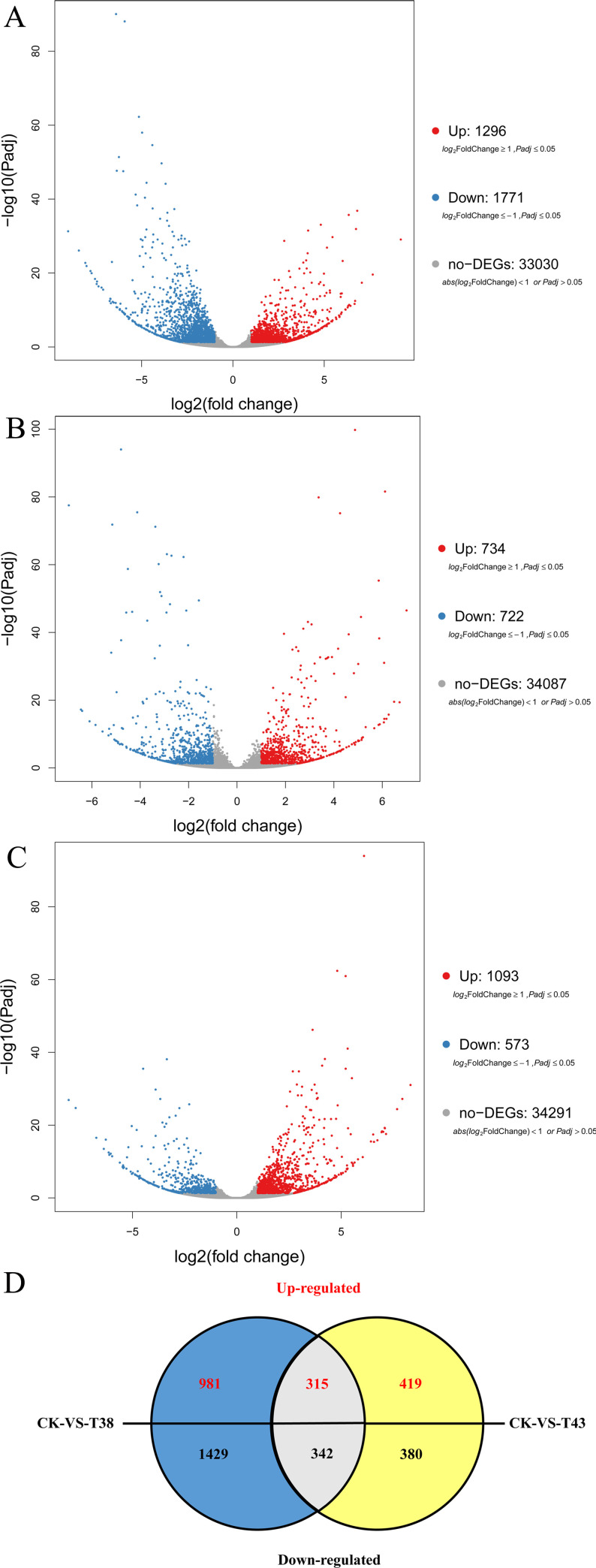
DEGs in groups T38 vs CK, T43 vs CK, and T43 vs T38. Volcano plots of DEGs in group T38 vs CK (A), group T43 vs CK (B), and group T43 vs T38 (C). (D) Venn diagram depicting the numbers of DEGs found in groups T38 vs CK and T43 vs CK.

To identify the DEGs in the T38 and T43 samples compared with the CK samples, we calculated the transcript abundance of genes with FPKM method [31] and identified the DEGs by setting a threshold of |log2 fold-change| > 1 and *p*-value < 0.05. As shown in Fig 1 and S1 Fig, there were 3,067 DEGs (1,296 and 1,771 up- and down-regulated genes, respectively) in T38 vs CK and 1,456 DEGs (734 and 722 up- and down-regulated genes, respectively) in T43 vs CK. In these two groups of DEGs, 315 genes were up-regulated, and 342 genes were down-regulated in common (Fig 1D). In comparison to the T38, 1093 DEGs were up-regulated and 573 DEGs were down-regulated in the T43 treatment samples (Fig 1C and S1C Fig).

### GO functional enrichment analyses of DEGs

**Fig 2:**
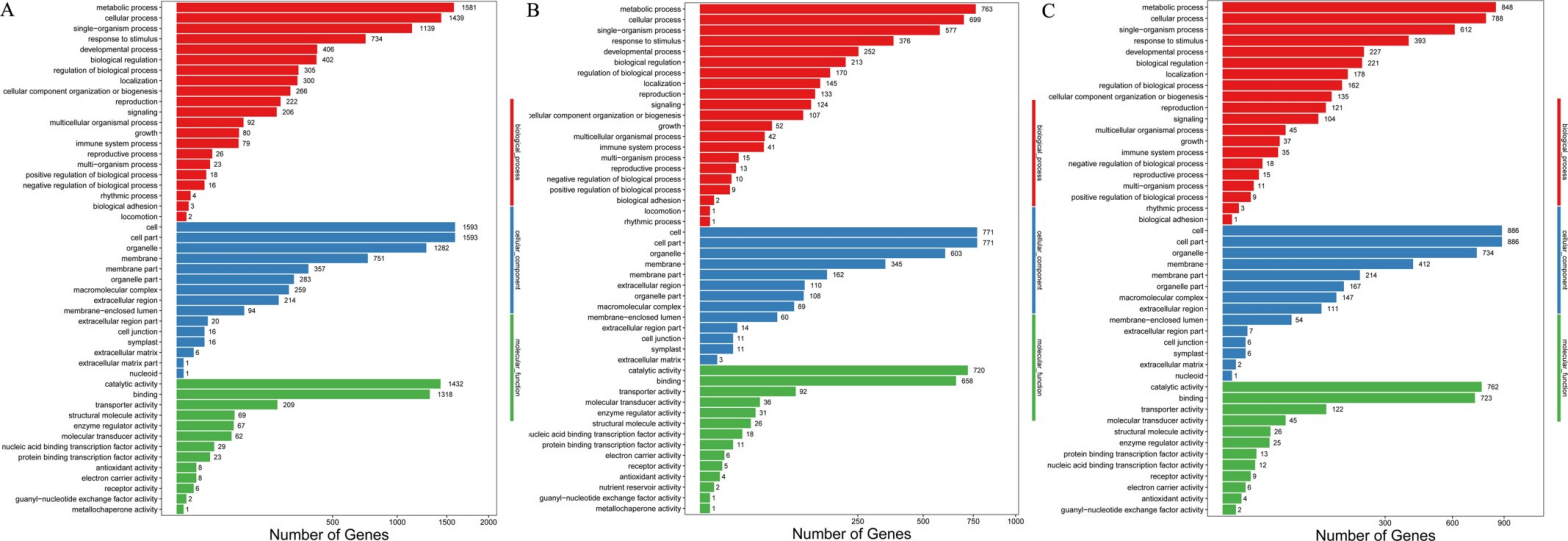
GO classification of DEGs. GO classification of DEGs in group T38 vs CK (A), group T43 vs CK (B) and group T43 vs T38 (C). The DEGs are summarized in three main categories: biological process, cellular component and molecular function. The X-axis indicates the number of genes and Y-axis indicates the GO terms.

To illustrate the DEGs detected in the T38, T43, and CK samples, their functional classes were evaluated by GO enrichment analyses. DEGs were divided into three major functional categories: molecular function, cellular component, and biological process. The DEGs in the T38 vs CK group were mainly clustered in metabolic process (1,581 unigenes), cellular process (1,439 unigenes), single-organism process (1,139 unigenes), and response to stimulus (734 unigenes) in the biological process category. Cell (1,593 unigenes), cell part (1,593 unigenes), organelle (1,282 unigenes), and membrane (751 unigenes) dominated the cellular component category. Catalytic activity (1,432 unigenes), binding (1,318 unigenes), and transporter activity (209 unigenes) were the top three terms in the molecular function category (Fig 2A). As shown in Fig 2B and 2C, GO functional enrichment analysis of the T43 vs CK group and T43 vs T38 group revealed a similar classification as the T38 vs CK group.

**Fig 3:**
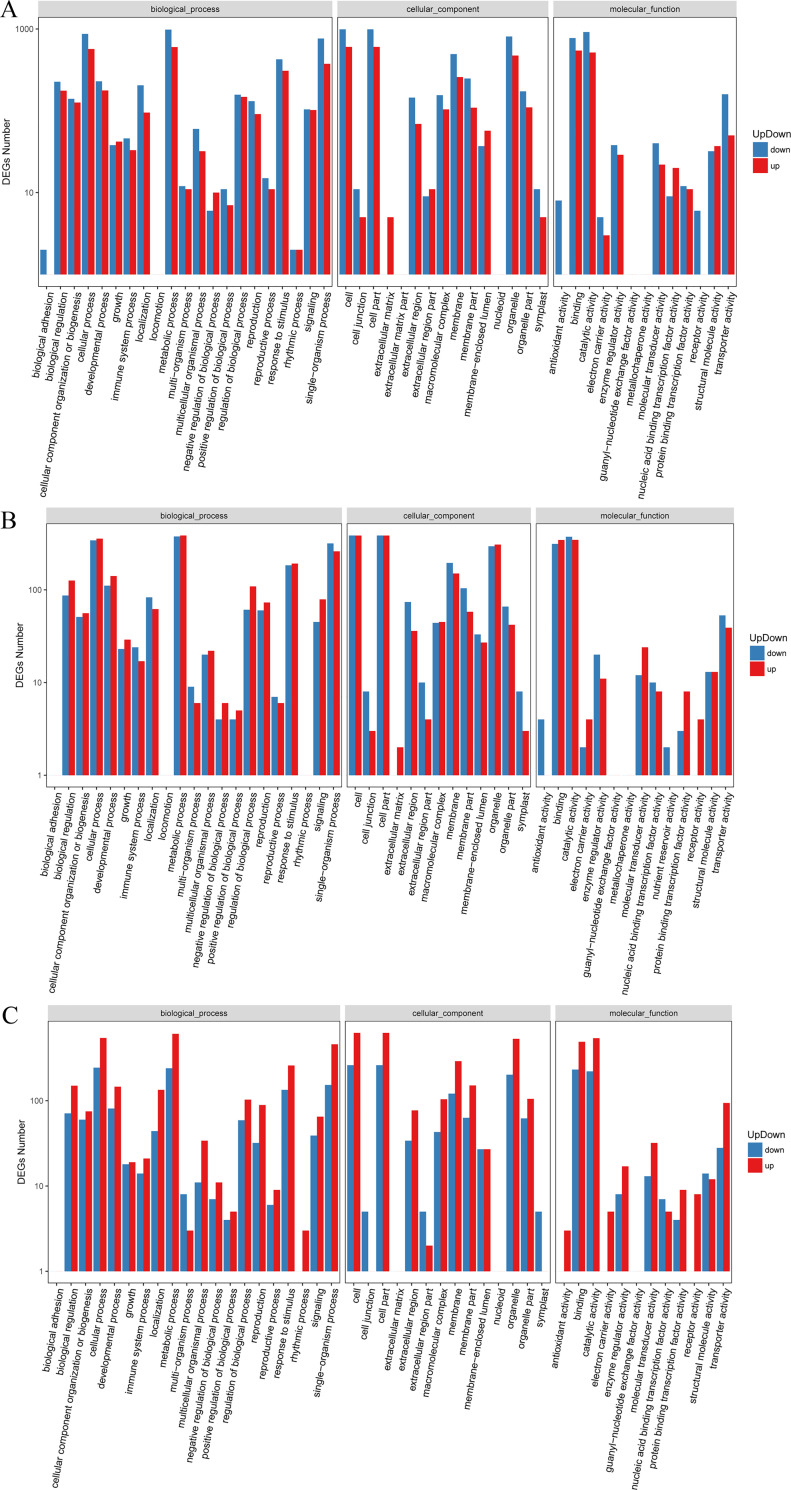
GO classification of up- and down-regulated DEGs. GO classification of up- and down-regulated DEGs in group T38 vs CK (A), group T43 vs CK (B), and group T43 vs T38 (C). The DEGs are summarized in three main categories: biological process, cellular component and molecular function. The X-axis indicates the GO terms and Y-axis indicates the number of genes.

To further explore the classification of the DEGs, up- and down-regulated DEGs were assigned to one or more GO terms. The up- and down-regulated genes in the T38 vs CK group, T43 vs CK group, and T43 vs T38 showed similar enrichment in GO analysis. Cellular process, metabolic process, single-organism process, and response to stimulus were the most enriched terms in the biological process category. Cell, cell part, and organelle were highly clustered terms in the cellular component category. In the molecular function category, binding, catalytic activity, and transporter activity were significantly higher than the other terms (Fig 3). As shown in Figs 2 and 3, the classifications of up- and down- regulated genes in the three groups were consistent with the DEG classifications.

### KEGG pathway enrichment analyses of DEGs

**Fig 4:**
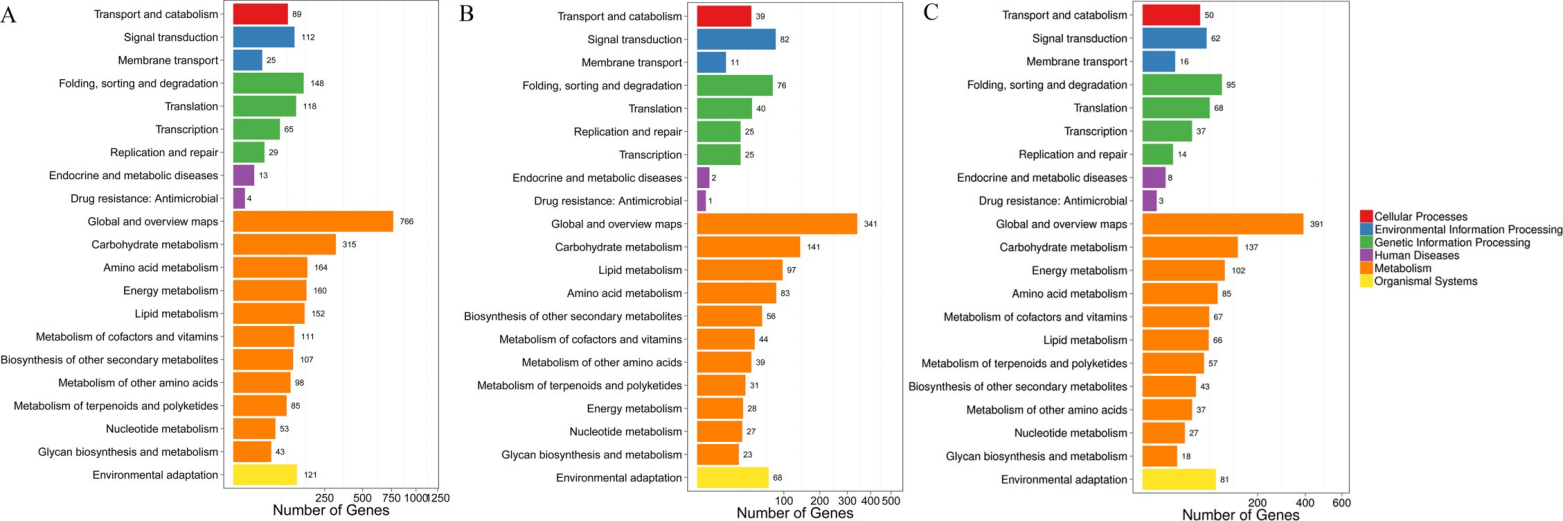
KEGG biological pathway classification. (A) KEGG biological pathway classification in group T38 vs CK. (B) KEGG biological pathway classification in group T43 vs CK. (C) KEGG biological pathway classification in group T43 vs T38. The X axis represents the proportion of genes, and the Y axis represents the KEGG function classification.

The DEGs were mapped to the reference pathways in the KEGG database. DEGs were classified into six categories: cellular processes, environmental information processing, genetic information processing, human diseases, metabolism, and organismal systems. Among them, the ‘metabolism’ accounted for the largest proportion of enriched genes (Fig 4 and S2 Fig). In the T38 vs CK group, T43 vs CK group, and T43 vs T38 group, up- and down-regulated DEGs were significantly enriched in the ‘biosynthesis of secondary metabolites’ and ‘metabolic pathways’, showing consistent results with KEGG pathway enrichment analyses (Fig 4 and S3 Fig).

### DEGs identified as transcription factors in eggplant leaves

**Fig 5:**
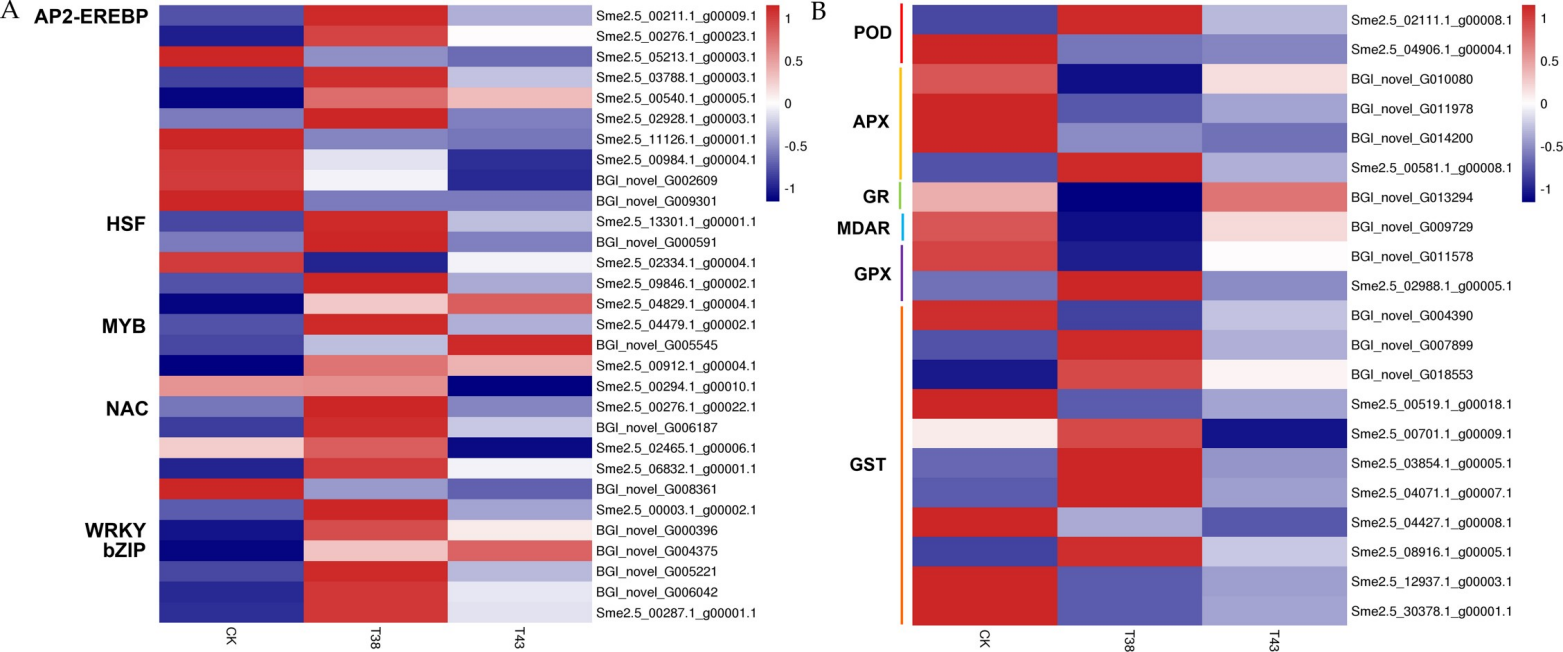
Heat map representation of the expression patterns of TFs (A) and genes related to antioxidant enzyme systems (B) in eggplant leaves. AP2-EREBP, APETALA2-ethylene-responsive element binding protein; Hsf, heat shock transcription factor; bZIP, basic region/leucine zipper; POD, peroxidase; APX, ascorbate peroxidase; GR, glutathione reductase; MDAR, monodehydroascorbate reductase; GPX, glutathione peroxidase; and GST, glutathione S-transferase. Columns in the heat map represent samples collected under different temperature treatments. The color scale on the right represents the log-transformed FPKM value.

TFs play important roles in several processes in plant development, including abiotic stress responses [3]. To investigate the effect of heat stress on TFs, we measured the expression levels of them. There were 59 DEGs involved in 20 transcription factor families (Table 3). In this study, five AP2-EREBP (APETALA2- ethylene-responsive element binding protein) genes were up-regulated and three were down-regulated in the T38 vs CK group, whereas three genes were up-regulated and five genes were down-regulated in the T43 vs CK group. Among them, six genes were co-regulated in the two groups, showing similar patterns. Five Hsf genes were differentially expressed in eggplant. Three of these genes were up-regulated and one gene was down-regulated in the T38 vs CK groups. Two genes were up-regulated in the T43 vs CK group. The expression levels of four MYB genes were significantly changed with different treatments. Two MYB genes (Sme2.5_04479.1_g00002.1 and Sme2.5_00912.1_g00004.1) were commonly up-regulated in the two groups. Five and three genes belonging to the NAC family showed differential expression in two groups. Only one WRKY (BGI_novel_G000396) gene and four basic region/leucine zipper genes (BGI_novel_G004375, BGI_novel_G005221, BGI_novel_G006042, and Sme2.5_00287.1_g00001.1) were significantly up-regulated in both groups (Table 3 and Fig 5A).

**Table 3:** The number of DEGs identified as transcription factors in eggplant leaves.

Category	Total	T38 vs CK		T43 vs CK	
		up-regulated	down-regulated	up-regulated	down-regulated
AP2-EREBP	10	5	3	2	5
ARF	4	2	2	0	0
Alfin-like	2	0	2	0	0
C2C2-CO-like	2	0	2	0	0
C2C2-Dof	3	2	1	0	0
C2C2-YABBY	1	0	1	0	0
C3H	2	2	0	0	0
FAR1	2	1	1	0	0
GRAS	1	1	0	1	0
Hsf	5	3	1	2	0
MYB	4	2	0	3	1
NAC	6	4	1	1	2
SBP	1	0	0	0	1
TAZ	1	1	0	1	0
Trihelix	3	3	0	1	0
ULT	1	1	0	0	0
WRKY	1	1	0	1	0
bHLH	5	3	1	0	2
bZIP	4	4	0	4	0
mTERF	1	1	0	0	0

### Expression of genes related to antioxidant enzyme systems and detoxication

To investigate the effect of heat stress on antioxidant enzyme systems, we analyzed the expression of antioxidant enzyme system-related genes. As shown in S3 Table, 30 GO terms with 118 genes displayed differential expression in the T38 vs CK group. Meanwhile, there were 23 GO terms with 57 genes involved in antioxidant enzyme systems in the T43 vs CK group. In the T38 vs CK group, most antioxidant genes, which encode peroxidase (POD), ascorbate peroxidase (APX), glutathione reductase (GR), monodehydroascorbate reductase (MDAR), and glutathione peroxidase (GPX), exhibited significantly low expression, except for three genes (Sme2.5_02111.1_g00008.1, Sme2.5_00581.1_g00008.1, and Sme2.5_02988.1_g00005.1) encoding POD, APX, and GPX respectively. Three of the down-regulated genes (Sme2.5_04906.1_g00004.1, BGI_novel_G011978, and BGI_novel_G014200) above also exhibited low expressed in the T43 sample compared to the CK sample. Five genes encoding glutathione *S*-transferase (GST) were up-regulated and four genes were down-regulated in the T38 vs CK group. Meanwhile, six GST genes were highly expressed and two genes showed low expression in the T43 vs CK group. Among them, four genes were commonly down-regulated, and two genes were up-regulated in the two groups (Fig 5B).

**Fig 6:**
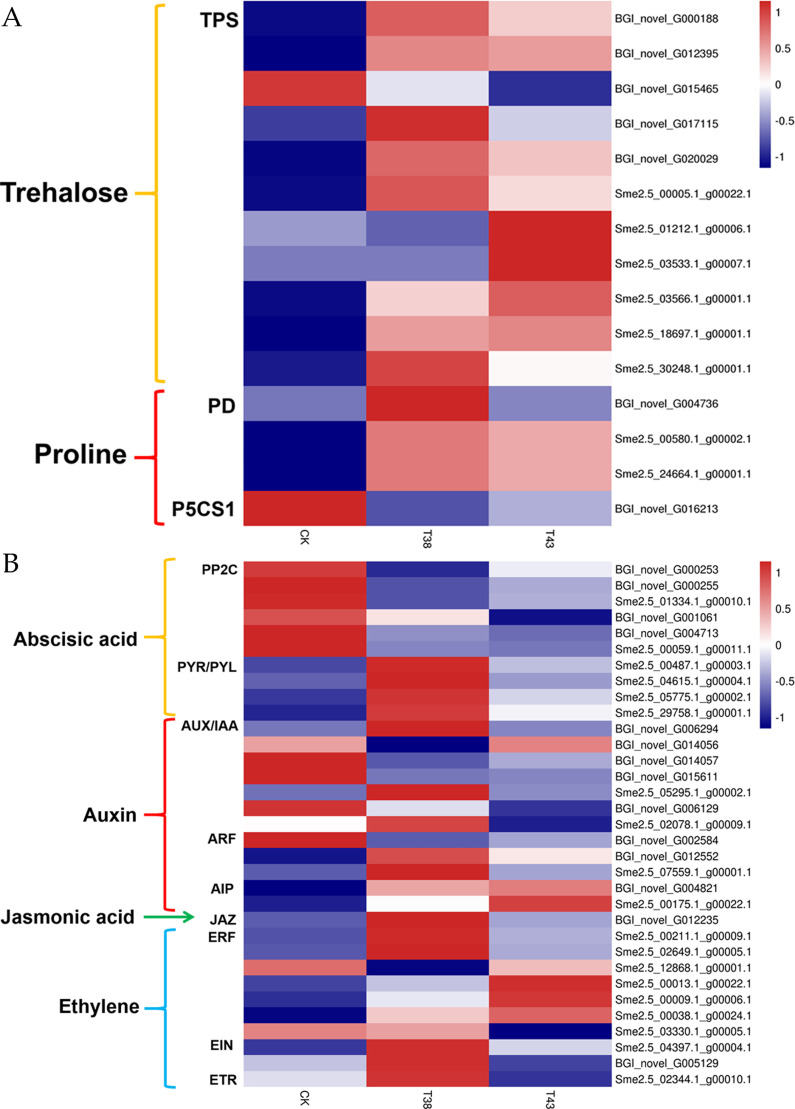
Heat map representation of the expression patterns of genes related to detoxication (a) and phytohormone (b) in eggplant leaves. TPS, trehalose-phosphatase; PD, proline dehydrogenase; P5CS1, delta1-pyrroline-5-carboxylate synthase 1; PP2C, protein phosphatase 2C; PYR/PYL, abscisic acid receptor PYR/PYL family; AUX, auxin influx carrier; IAA, auxin-responsive protein IAA; ARF, auxin response factor; AIP, auxin-induced protein; JAZ, jasmonate ZIM domain-containing protein; ERF, ethylene-responsive transcription factor; EIN, ethylene-insensitive protein; and ETR, ethylene receptor. Columns in the heat map represent samples collected under different temperature treatments. The color scale on the right represents the log-transformed FPKM value.

Plants display toxic symptoms under adverse conditions [37]. To explore the effect of heat stress on detoxication, we performed GO analysis to evaluate the enrichment of the DEGs. There were 3 GO terms involved in detoxication process, which included 12 and 10 DEGs in the T38 vs CK groups and T43 vs CK groups, respectively (S3 Table). Some osmotic factors, such as trehalose and proline, play roles in detoxification through oxidation [37]. As shown in S3 Table, four GO terms contained five and six DEGs in the T38 vs CK and T43 vs the CK groups, respectively. After assessing the expression pattern of trehalose-related genes, we found that six DEGs encoding trehalose-phosphatase were all up-regulated in the T38 vs CK group. Eight genes encoding trehalose-6-phosphate synthase (TPS) were up-regulated and only one gene (BGI_novel_G015465) was down-regulated in the T43 vs CK groups (Fig 6A). As shown in Fig 6A, proline-related genes encoding proline dehydrogenase (PD) and delta1-pyrroline-5-carboxylate synthase 1 (P5CS1) were all significantly up-regulated in T38 and T43 samples compared with CK.

### Expression of genes associated with phytohormones

Endogenous hormones respond to exogenous stimuli and regulate signaling in plants. To determine the functions of plant hormones in heat stress, we assessed the expression patterns of hormone-related genes encoding receptors and response factors.

As shown in S3 Table, there were 9 and 10 GO terms involved in abscisic acid process, which included 71 and 45 DEGs in the T38 vs CK groups and T43 vs CK groups, respectively. Three genes encoding protein phosphatase 2C (PP2C) showed low expression in the T38 sample, whereas four genes showed low expression in the T43 sample compared with in the CK group. Four genes encoding the abscisic acid receptor (PYR/PYL) were highly expressed in the T38 sample compared with the CK group. Among them, two genes were also highly expressed in the T43 sample (Fig 6B).

In auxin-related processes, 12 and 9 GO terms that included 44 and 25 DEGs displayed significant differences in the T38 vs CK groups and T43 vs CK groups, respectively (S3 Table). Five genes encoding auxin influx carrier/auxin-responsive protein IAA (AUX/IAA) were differentially expressed in the T38 vs CK groups. Among them, the expression levels of three genes were down-regulated and two genes were up-regulated. Two up-regulated genes and one down-regulated gene encoded the auxin response factor (ARF) in the T38 vs CK groups. Two genes encoding AUX/IAA were down-regulated, whereas two genes encoding auxin-induced protein were regulated in the T43 vs CK groups (Fig 6B).

In ethylene-related processes, 8 and 7 GO terms that included 31 and 21 DEGs displayed significant differences between the T38 vs CK groups and T43 vs CK groups, respectively (S3 Table). Most genes encoding ethylene-responsive transcription factor (ERF) were up-regulated except for two genes in the two groups. Expression of ethylene-insensitive protein (EIN) genes was significantly up-regulated in the T38 vs CK group and down-regulated in the T43 vs CK group. A gene encoding the ethylene receptor (ETR) was significantly down-regulated in the T43 vs CK group (Fig 6B).

In jasmonic acid-related processes, 8 and 6 GO terms that included 26 and 11 DEGs displayed significant differences in the T38 vs CK groups and T43 vs CK groups, respectively (S3 Table). Expression of the jasmonate ZIM domain-containing gene (BGI_novel_G012235) was significantly up-regulated after exposure to high temperature (Fig 6B).

### Expression analyses of heat stress-related genes

**Fig 7:**
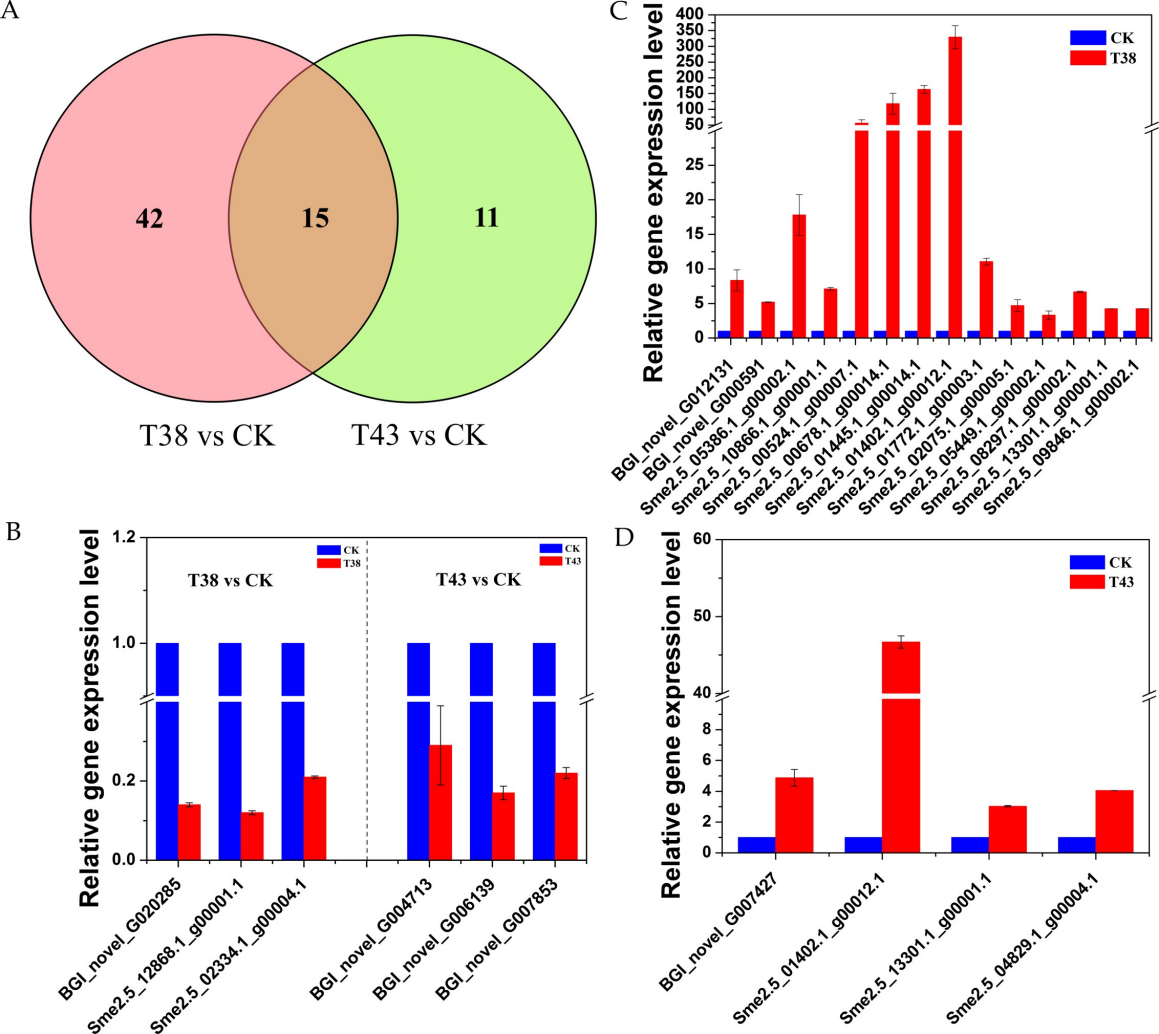
Expression analyses of eggplant’s heat stress related genes. (a) Venn diagram depicting the numbers of heat stress related DEGs found in groups T38 vs CK and T43 vs CK. (b) Down-regulated genes in groups T38 vs CK and T43 vs CK. (c) Up-regulated genes in group T38 vs CK. (d) Up-regulated genes in group T43 vs CK. X-axis stands for different gene id. Y-axis stands for the relative expression level. The values were normalized against the levels of *SmEF1a* as a control.

To identify heat stress-related genes among the DEGs, we further investigated the GO terms in the biological process category. Four terms, response to heat (GO:0009408), heat acclimation (GO:0010286), regulation of cellular response to heat (GO:1900034), and cellular response to heat (GO:0034605), with 57 DEGs enriched were significantly related to heat stress in the T38 vs CK group. In the T43 vs CK group, cellular response to heat (GO:0034605), heat acclimation (GO:0010286), and response to heat (GO:0009408), with 26 DEGs were selected. Among them, 15 genes were differentially expressed in common between the groups (Fig 7A and S4 Fig). There were 34 and 9 DEGs encoding HSPs in the T38 vs CK group and T43 vs CK group, respectively (S4 Fig). As shown in Fig 5B and Table 4, five Hsfs were differentially expressed in the two groups. Sme2.5_13301.1_g00001.1, which encodes heat shock transcription factor B2a, was up-regulated commonly in two groups. Additionally, there were other related genes, including APX, PP2C, abscisic acid, ERF, early light-induced protein (ELIP), glucose-1-phosphate adenylyltransferase family protein, glyceraldehyde-3-phosphate dehydrogenase, phosphoglycerate kinase, galactinol synthase, etc. BGI_novel_G020285 and BGI_novel_G004713, which encode protein phosphatase 2C, were down-regulated in the T38 vs CK group and T43 vs CK group, respectively. Two genes (Sme2.5_19309.1_g00001.1 and BGI_novel_G020064), which encode glyceraldehyde 3-phosphate dehydrogenase, were commonly down-regulated in two groups. BGI_novel_G010489 (annexin 2) and BGI_novel_G001773 (annexin 4) were down-regulated in group T38 vs CK and group T43 vs CK, respectively. BGI_novel_G014702, which was a glucose-1-phosphate adenylyltransferase family protein, was significantly down-regulated in two groups. There were one and two DEGs encoding annexin were down-regulated in group T38 vs CK and group T43 vs CK, respectively. Among them, BGI_novel_G010489 (annexin 2) was commonly down-regulated in two groups.

**Table 4:** Heat stress related genes id, log2 fold change in RNA-Seq and their descriptions.

GENE ID	log2 fold change	description
BGI_novel_G000591	2.25(T38 vs CK)	Heat shock factors
Sme2.5_13301.1_g00001.1	1.92(T38 vs CK)	Heat shock transcription factor B2a
	1.08(T43 vs CK)	
Sme2.5_09846.1_g00002.1	2.02(T38 vs CK)	Heat shock factors, HsfA5
Sme2.5_02334.1_g00004.1	-1.87(T38 vs CK)	Heat shock factors, Hsf3, HsfA1b
Sme2.5_01402.1_g00012.1	6.74(T38 vs CK)	HSP20 family protein
	4.61(T43 vs CK)	
Sme2.5_02075.1_g00005.1	2.64(T38 vs CK)	HSP20 family protein
Sme2.5_05386.1_g00002.1	4.32(T38 vs CK)	HSP20 family protein
Sme2.5_10866.1_g00001.1	2.93(T38 vs CK)	HSP20 family protein
Sme2.5_00524.1_g00007.1	4.99(T38 vs CK)	HSP20 family protein
Sme2.5_00678.1_g00014.1	4.16(T38 vs CK)	HSP20 family protein
Sme2.5_01445.1_g00014.1	2.91(T38 vs CK)	HSP20 family protein
BGI_novel_G012131	2.28(T38 vs CK)	HSP40 family protein, DnaJ homolog subfamily A member 2
Sme2.5_05449.1_g00002.1	3.02(T38 vs CK)	HSP70 family protein
Sme2.5_08297.1_g00002.1	2.68(T38 vs CK)	HSP70 family protein
Sme2.5_01772.1_g00003.1	3.07(T38 vs CK)	HSP100 family protein
BGI_novel_G020285	-4.40(T38 vs CK)	protein phosphatase 2C
Sme2.5_12868.1_g00001.1	-2.12(T38 vs CK)	EREBP-like factor
Sme2.5_04829.1_g00004.1	1.71(T43 vs CK)	heat shock transcription factor C1
BGI_novel_G006139	-2.41(T43 vs CK)	HSP20 family protein
BGI_novel_G007853	-2.47(T43 vs CK)	HSP20 family protein
BGI_novel_G007427	2.11(T43 vs CK)	HSP20 family protein
BGI_novel_G004713	-5.31(T43 vs CK)	protein phosphatase 2C

Two and one genes, which encode early light-induced protein, were down-regulated in group T38 vs CK and group T43 vs CK, respectively. Similarly, Sme2.5_00065.1_g00018.1 (ELIP1) was down-regulated in two groups. Sme2.5_01945.1_g00010.1, which encodes phosphoglycerate kinase, was significantly down-regulated in two groups (S4 Fig).

To further validate the DEGs obtained in RNA-Seq, 22 genes were selected (Table 4) and their expression patterns were examined by qRT-PCR. As shown in Fig 6B–6D, the qRT-PCR results agreed with the Illumina sequencing results.

## Discussion

Plant productivity and quality are challenged by environmental stresses, including heat stress and drought. Plants have evolved a flexible and diverse adaptation mechanism for surviving from stress conditions [38]. They can sense signals from outside of cells and deliver this information into cells for cascade amplification. Plants regulate their growth and development to respond to different environments. Plants respond to heat stress through a complex network; however, the related transcriptional profiles have not been widely studied in eggplant. Here, we cultivated the heat-tolerant eggplant variety Tewangda and performed RNA-Seq to characterize the genome-wide transcriptome dynamics and differential expression of genes during heat stress.

The Q20 of clean reads in three samples (Table 1) reflected the sequencing reliability, which indicated that the sequencer, sequencing reagents, and samples’ quality were reliable. The average mapping ratio of samples to the reference genome was 82.68% (Table 2). The mapping ratios were comparable among the different samples. To further identify DEGs in T38 and T43 samples compared with the CK sample, we displayed the DEGs in a volcano plot, Venn plot, and MA-plot (Fig 1 and S1 Fig). There was a large difference in the number of DEGs between the T38 vs CK group (1, 296/1, 771 genes up-/down-regulated) and T43 vs CK group (734/722 genes up-/down-regulated). These results indicate that there are differences in the regulation mechanisms under different temperature treatments in eggplant.

TFs play vital roles in abiotic and biotic stresses. They modulate gene expression by binding cis-elements of given genes under different biological processes. Ethylene-responsive factor/ dehydration-responsive element binding (ERF/DREB), as a subfamily of AP2/EREBP family, involves in many abiotic stresses. Overexpression of *CmDREB6* in chrysanthemum (*Chrysanthemum morifolium*) enhanced tolerance to heat and promoted the expression of *CmHsfA4*, *CmHsp90*, *CmSOD*, and *CmCAT* [39]. DREB2A and DREB2C regulated heat tolerance by binding to promoter of *HsfA3* in *Arabidopsis* [40, 41]. Overexpression of *TaERF3* in wheat enhanced tolerance to drought and salt stress, while virus-induced gene silencing (VIGS) of *TaERF3* were more sensitive to salt and drought [42]. In our research, three genes (Sme2.5_00211.1_g00009.1, Sme2.5_00276.1_g00023.1, and Sme2.5_00540.1_g00005.1) encoding ERF/DREB were significantly up-regulated and three genes (Sme2.5_05213.1_g00003.1, Sme2.5_11126.1_g00001.1, and BGI_novel_G002609) encoding ERF/DREB were down-regulated in T38 vs CK and T43 vs CK. Among them, *ERF1* (Sme2.5_00540.1_g00005.1) up-regulated more than 10 fold in two groups (Fig 5A). These results indicated genes in the same family may play different roles under heat stress. As one of the largest TF families in plants, MYB proteins involve in plant growth, development, stress responses, etc. Two MYB genes were up-regulated in T38 vs CK group, while three MYB genes were up-regulated and one gene was down-regulated in T43 vs CK group. Among them, Sme2.5_04479.1_g00002.1 and Sme2.5_00912.1_g00004.1 were commonly up-regulated in two groups, which indicated that MYB TFs response to heat stress of eggplant (Fig 5A). NAC transcription factors were reported as key regulators in response to abiotic stresses. The *RESPONSIVE TO DEHYDRATION 26* (*RD26*) was the first identified gene mediating cross-talk between ABA and JA signaling during stress responses in *Arabidopsis* [43]. The wheat NAC TF TaNAC2L promotes heat stress tolerance in transgenic *Arabidopsis* [44]. Four genes were up-regulated and one gene was down-regulated in T38 vs CK. One gene was up-regulated and two genes were down-regulated in T43 vs CK. The genes Sme2.5_06832.1_g00001.1 and BGI_novel_G008361 expressed in the same pattern in the two groups. Four genes (BGI_novel_G004375, BGI_novel_G005221, BGI_novel_G006042, and Sme2.5_00287.1_g00001.1) encoding basic region/leucine zipper proteins were significantly up-regulated in both groups (Fig 5A). As previously reported, overexpression of a stress-responsive NAC gene *SNAC3* and *NTL3* increased heat tolerance in rice [45, 46]. OsNTL3 regulated heat stress response by binding to *OsbZIP74* promoter. Interestingly, the induction of *OsNTL3* expression was depended on OsbZIP74 [46]. Thus, the NAC genes and bZIP genes in our research may exist the interaction and the detail needs to be further investigated.

Plants are protected from heat induced oxidative stress by synthesis of various enzymatic ROS scavenging [47]. Expression levels of 57 genes related to antioxidant enzyme systems displayed significantly changed in this research. 21 of the 57 genes encode POD, APX, GR, MDAR, GPX and GST. As TFs always influence the stress responses of plant through regulating the accumulation of antioxidant enzyme, they may be the downstream genes of differently expressed TFs mentioned above. In addition to antioxidant enzymes, trehalose and proline function as osmotic regulators in plant [37]. Trehalose, a non-reducing disaccharide widely existing in flowering plant, participates in seed development, vegetative growth, flowering, and stress response. Trehalose is synthetized from trehalose-6-phosphate’s conversion by catalysis of TPS and trehalose-6-phosphate phosphatase (TPP) [48]. 11 genes encoding TPS showed significantly different expression in our research (Fig 6A), which indicates that trehalose biosynthesis may be effected by *TPS* genes to adapt heat stress conditions in eggplant. Meanwhile, proline, as an adaptive response to stress conditions, also plays an important role [49]. The expression levels of three *PD* genes and *P5CS1* gene were changed under different heat treatments.

Plant hormone participates in regulation of stress responses in plant. In the present study, hormone-related genes encoding receptors and response factors were significantly changed. *PP2C*, *PYR/PYL*, *AUX/IAA*, *ARF*, *AIP*, *JAZ*, *ERF*, *EIN*, and *ETR* genes that belong to biosynthesis/metabolism pathways of ABA, Auxin, JA, Eth showed different expression patterns (Fig 6B). Genomic, transcriptome, and proteome data of plants under heat shock treatment revealed that many genes, particularly heat shock factors and heat shock proteins, participated in the heat stress response of protein folding [50]. Among the heat stress-related genes in eggplant, 34 and 9 DEGs encode HSPs in the T38 vs CK group and T43 vs CK group, respectively (S4 Fig and Table 4). Heat stress often causes changes in protein conformations and triggers HSP accumulation. HSPs facilitate protein refolding and enzyme and membrane stabilization, and overexpression of these HSPs can increase the heat tolerance of plants [51, 52]. sHSPs (small heat shock proteins) participate in various biological processes, including the cell cycle, cell differentiation, adaptation to stressful conditions, and apoptosis [53]. Sme2.5_01402.1_g00012.1, an sHSP, shows significant homology with at4g27670 in Arabidopsis. It was reported that the plastid metalloproteases FtsH6 and HSP21 (HSP21) jointly regulate thermomemory in *Arabidopsis* [54]. A new transcript, BGI_novel_G007427, is homologous to at1g06460, which encodes an α-crystallin domain-containing protein with homology to sHSPs and is down-regulated by FPF1 overexpression, long days, floral induction, and gibberellin in Arabidopsis wild-type [55]. Sme2.5_05449.1_g00002.1 and Sme2.5_08297.1_g00002.1 belong to the HSP70 family. HSP70s were reported to accumulate during heat stress and are essential for general cellular functions because of their roles in protein homeostasis [56]. ClpB/HSP100 proteins play a crucial role in thermotolerance induction in bacteria, yeasts, and plants [57]. Sme2.5_01772.1_g00003.1, a ClpB/HSP100 protein, is homologous to AtHOT1 in Arabidopsis. HOT1 mutants with impaired AtHSP101/ClpB gene function cannot survive at high temperatures [58].

Hsfs, which were divided into class A, B, and C, are upstream direct regulators of Hsps [59]. Sme2.5_02334.1_g00004.1 and Sme2.5_09846.1_g00002.1 are class A Hsfs. In tomato, SlHsfA1 was first identified as a ‘master regulator’ in heat stress response. Overexpression of SlHsfA1 resulted in better tolerance to heat stress, whereas co-suppression lines were more sensitive to heat stress [60]. In Arabidopsis, the transcription factors HsfA1abd and e were constitutively expressed and were responsible for triggering the heat stress response. The interaction between AtHsfA1 and AtHsfA2 activates the expression of downstream heat stress response genes [61]. Sme2.5_13301.1_g00001.1 belongs to the class B Hsf family. The role of class B Hsfs in plants are different. In tomato, HsfB1 represents a novel type of coactivator that cooperates with class A Hsfs [62]. In Arabidopsis, AtHsfB1 and AtHsfB2b repress Hsp and Hsf expression at high temperatures [63]. Overall, the response to heat stress in eggplant involves a complicated network and numerous regulation processes.

Hsfs can exit the latent state under normal conditions and enter an activated state by induction of trimerization, high-affinity binding to DNA, and transcriptional activity upon heat stress in Arabidopsis. Under heat stress, HsfA1a, HsfA1b, and HsfA1d are activated and the expression of most HS-responsive genes is induced, including that of other Hsfs, other transcription factors, Hsps, and related genes. These transcription factors regulate additional target genes [61]. Our transcriptome data revealed numerous DEGs which may regulate heat stress responses in eggplant, a model similar to Arabidopsis. The detailed regulatory pattern of heat shock-related genes requires further verification.

## Supporting information

S1 Fig MA-plot of DEGs. (A) MA-plot of DEGs in group T38 vs CK. (B) MA-plot of DEGs in group T43 vs CK. The X-axis indicates A value, which is the average expression level after log2 conversion. The Y-axis indicates M value, which is the difference multiple after log2 conversion. Red dots represent the up-regulated DEGs. Blue dots represent the down-regulated DEGs. Grey dots represent the non-DEGs.
(DOC)

S2 Fig Top 20 pathways in KEGG enrichment by Qvalue. (A) Top 20 pathways in KEGG enrichment in group T38 vs CK. (B) Top 20 pathways in KEGG enrichment in group T43 vs CK. Rich Factor is the ratio of the differentially expressed number of genes in the pathway and the total number of genes in the pathway. The higher the Rich Factor, the higher the degree of enrichment. Qvalue is the *p*-value after the multiple hypothesis test correction, in the range of 0 to 1. The closer the Qvalue is to 0, the more significant the enrichment.
(DOC)

S3 Fig Up- and down-regulated DEGs number of the most enriched pathway. (A) DEGs number of the most enriched pathway in group T38 vs CK. (B) DEGs number of the most enriched pathway in group T43 vs CK. Red indicates the up-regulated DEGs. Blue indicates the down-regulated DEGs.
(DOC)

S4 Fig Heat map representation of the expression patterns of genes related to heat stress. Columns in the heat map represent samples collected under different temperature treatments. The color scale on the right represents the log-transformed FPKM value.
(DOC)

S1 Table Primers used for quantitative real-time PCR. (DOC)

S2 Table The prediction of new transcripts. (DOC)

S3 Table GO terms of DEGs related to antioxidant enzyme systems, detoxication, and phytohormone. (XLS)
