# Supplementary material for: Transcriptome profiling and gene expression analyses of eggplant (Solanum melongena L.) under heat stress
Source: PLoS One. 2020 Aug 11;15(8):e0236980. doi: 10.1371/journal.pone.0236980 (PMC7419001; doi:10.1371/journal.pone.0236980)

S1 Fig. MA-plot of DEGs. (A) MA-plot of DEGs in group T38 vs CK. (B) MA-plot of DEGs in group T43 vs CK. (C) MA-plot of DEGs in group T43 vs T38. The X-axis indicates A value, which is the average expression level after log2 conversion. The Y-axis indicates M value, which is the difference multiple after log2 conversion. Red dots represent the up-regulated DEGs. Blue dots represent the down-regulated DEGs. Grey dots represent the non-DEGs.


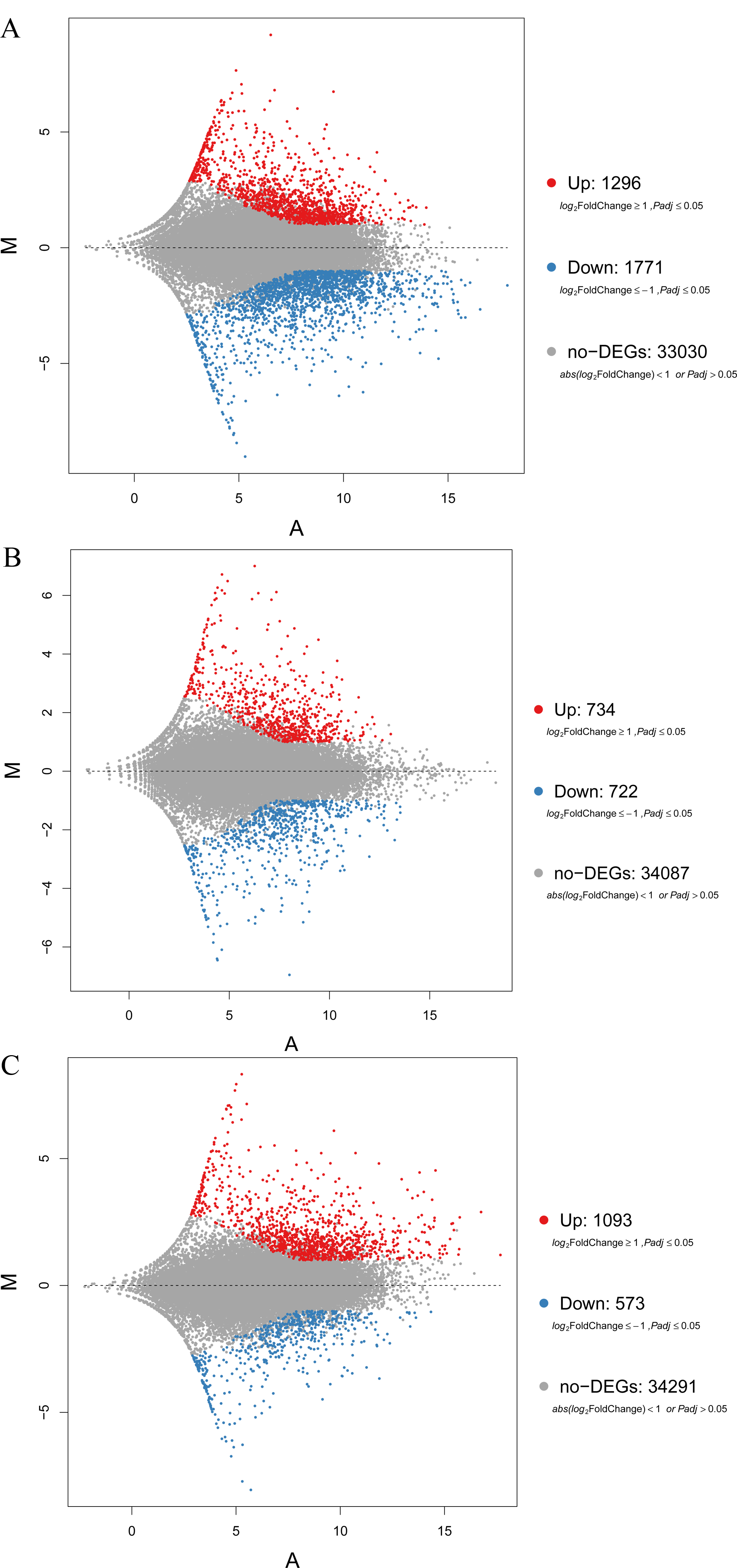

Supplement: S1 Fig — (A) MA-plot of DEGs in group T38 vs CK. (B) MA-plot of DEGs in group T43 vs CK. The X-axis indicates A value, which is the average expression level after log2 conversion. The Y-axis indicates M value, which is the difference multiple after log2 conversion. Red dots represent the up-regulated DEGs. Blue dots represent the down-regulated DEGs. Grey dots represent the non-DEGs. (DOC) [file pone.0236980.s001.doc]
