# Supplementary material for: Transcriptome profiling and gene expression analyses of eggplant (Solanum melongena L.) under heat stress
Source: PLoS One. 2020 Aug 11;15(8):e0236980. doi: 10.1371/journal.pone.0236980 (PMC7419001; doi:10.1371/journal.pone.0236980)

S2 Fig. Top 20 pathways in KEGG enrichment by Qvalue. (A) Top 20 pathways in KEGG enrichment in group T38 vs CK. (B) Top 20 pathways in KEGG enrichment in group T43 vs CK. (C) Top 20 pathways in KEGG enrichment in group T43 vs T38. Rich Factor is the ratio of the differentially expressed number of genes in the pathway and the total number of genes in the pathway. The higher the Rich Factor, the higher the degree of enrichment. Qvalue is the *p*-value after the multiple hypothesis test correction, in the range of 0 to 1. The closer the Qvalue is to 0, the more significant the enrichment.


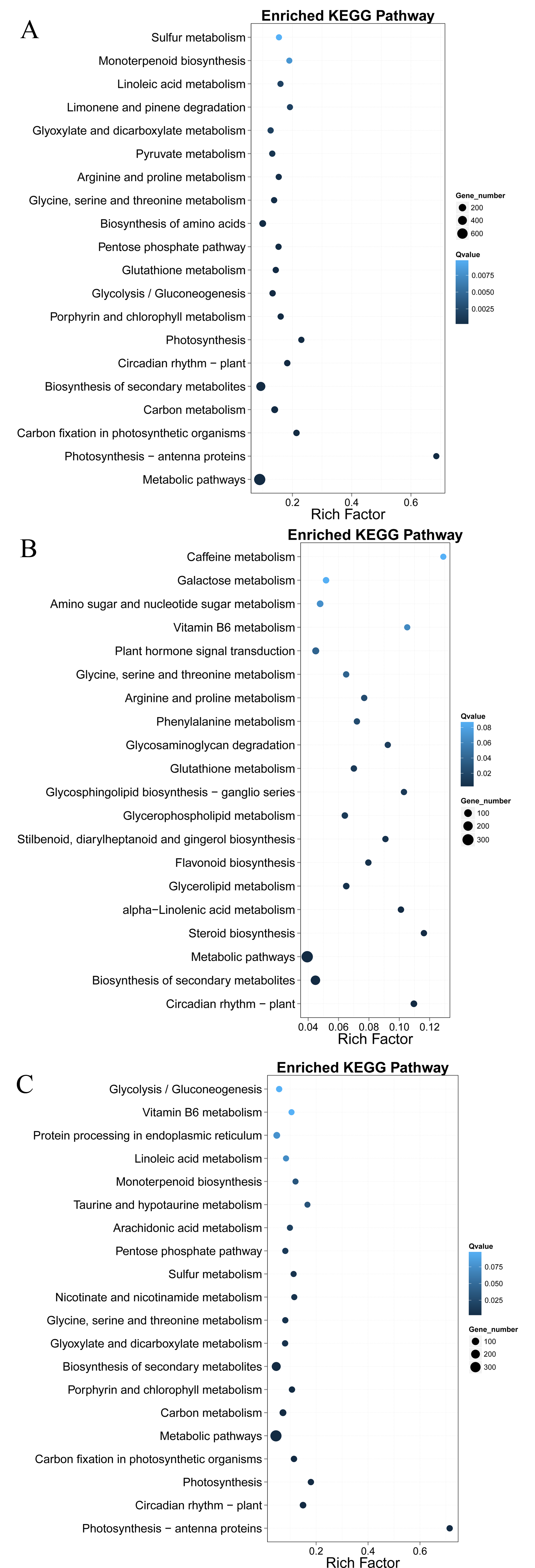

Supplement: S2 Fig — (A) Top 20 pathways in KEGG enrichment in group T38 vs CK. (B) Top 20 pathways in KEGG enrichment in group T43 vs CK. Rich Factor is the ratio of the differentially expressed number of genes in the pathway and the total number of genes in the pathway. The higher the Rich Factor, the higher the degree of enrichment. Qvalue is the p-value after the multiple hypothesis test correction, in the range of 0 to 1. The closer the Qvalue is to 0, the more significant the enrichment. (DOC) [file pone.0236980.s002.doc]
