# Supplementary material for: Transcriptome profiling and gene expression analyses of eggplant (Solanum melongena L.) under heat stress
Source: PLoS One. 2020 Aug 11;15(8):e0236980. doi: 10.1371/journal.pone.0236980 (PMC7419001; doi:10.1371/journal.pone.0236980)

S3 Fig. Up- and down-regulated DEGs number of the most enriched pathway. (A) DEGs number of the most enriched pathway in group T38 vs CK. (B) DEGs number of the most enriched pathway in group T43 vs CK. (C) DEGs number of the most enriched pathway in group T43 vs T38. Red indicates the up-regulated DEGs. Blue indicates the down-regulated DEGs.


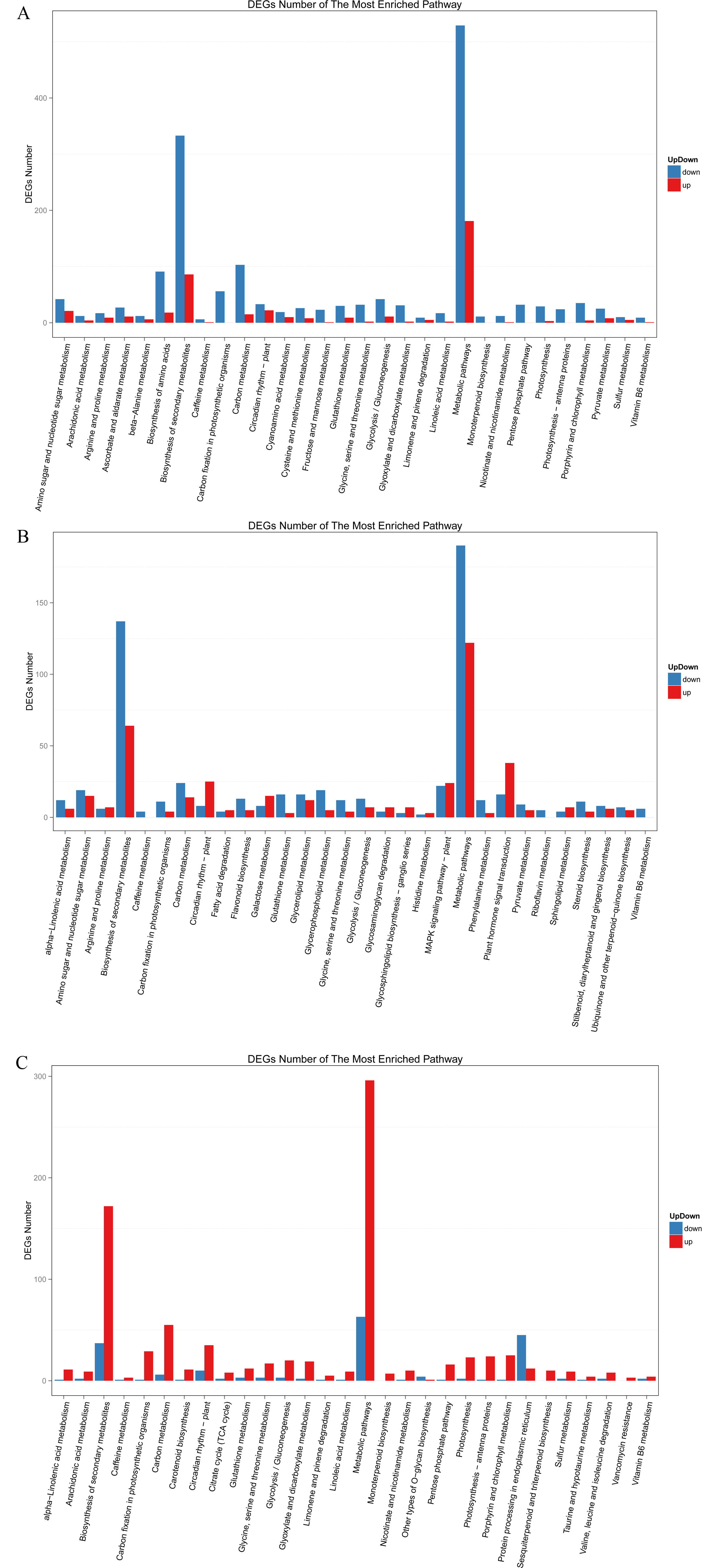

Supplement: S3 Fig — (A) DEGs number of the most enriched pathway in group T38 vs CK. (B) DEGs number of the most enriched pathway in group T43 vs CK. Red indicates the up-regulated DEGs. Blue indicates the down-regulated DEGs. (DOC) [file pone.0236980.s003.doc]
