# Supplementary material for: Transcriptome profiling and gene expression analyses of eggplant (Solanum melongena L.) under heat stress
Source: PLoS One. 2020 Aug 11;15(8):e0236980. doi: 10.1371/journal.pone.0236980 (PMC7419001; doi:10.1371/journal.pone.0236980)

S4 Fig. Heat map representation of the expression patterns of genes related to heat stress. Columns in the heat map represent samples collected under different temperature treatments. The color scale on the right represents the log-transformed FPKM value.


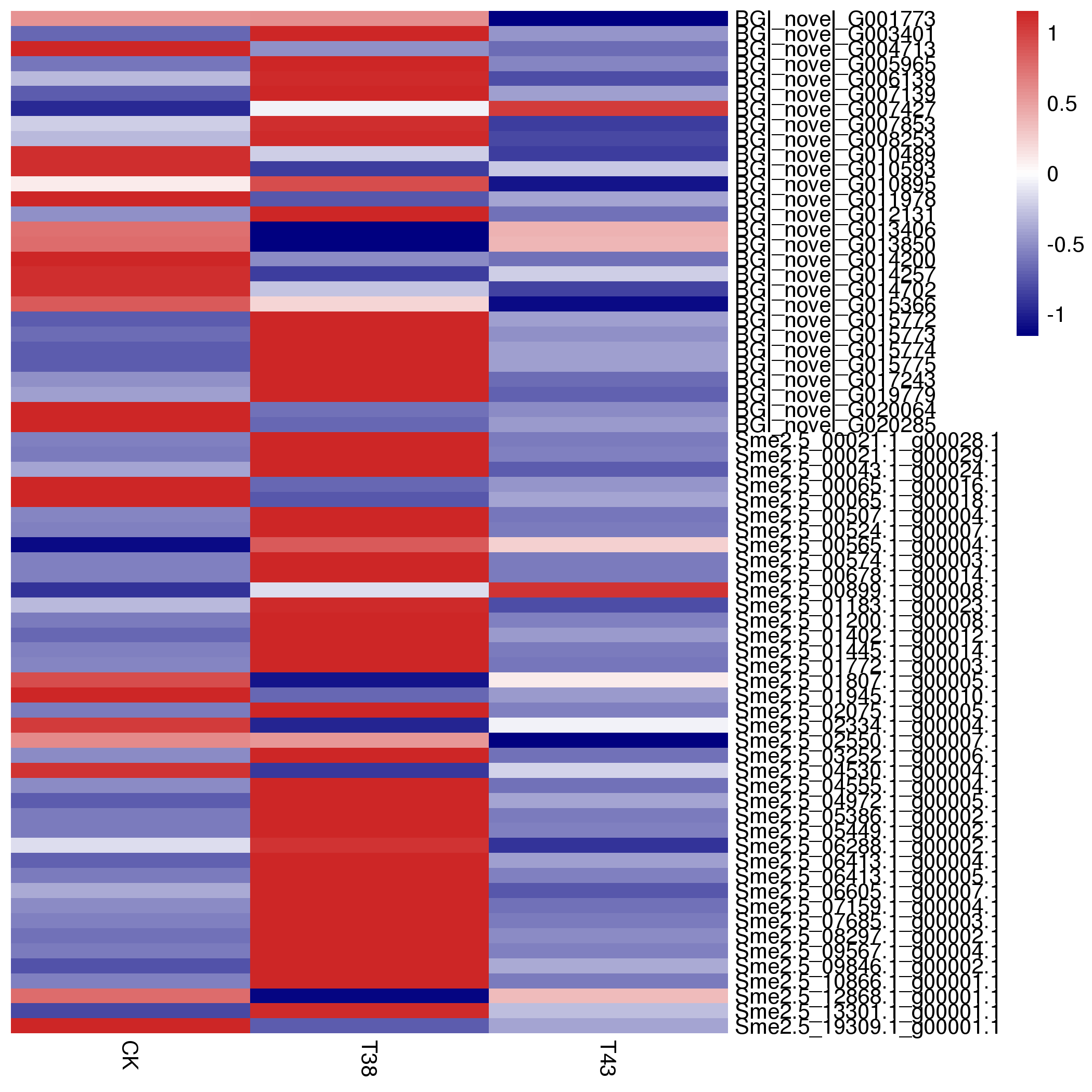

Supplement: S4 Fig — Columns in the heat map represent samples collected under different temperature treatments. The color scale on the right represents the log-transformed FPKM value. (DOC) [file pone.0236980.s004.doc]
