# Supplementary material for: Transcriptome profiling and gene expression analyses of eggplant (Solanum melongena L.) under heat stress
Source: PLoS One. 2020 Aug 11;15(8):e0236980. doi: 10.1371/journal.pone.0236980 (PMC7419001; doi:10.1371/journal.pone.0236980)
Supplement: S1 Table — (DOC) [file pone.0236980.s005.doc]

**S1 Table. Primers used for quantitative real-time PCR**

| **GENE ID** | **Sequence** |
| --- | --- |
| BGI_novel_G000591 | LP：5’-AAGGGGGAATAAGGGAGAAAATAAT-3‘ |
|  | RP：5’-TGGAATTAATCAAACTGTTGAAACG-3‘ |
| Sme2.5_13301.1_g00001.1 | LP：5’-GGTAGGGAAGGAGAAGAAGTAAAGG-3‘ |
|  | RP：5’-TGCAATTTCGCAAAGAAACGT-3‘ |
| Sme2.5_09846.1_g00002.1 | LP：5’-CCATTCACAGTCATAGTCACCCTC-3‘ |
|  | RP：5’-AGACATTAGCCTCAAGCCCAGA-3‘ |
| Sme2.5_02334.1_g00004.1 | LP：5’- GATACGGATGAGATTGGTTCCCTAG-3‘ |
|  | RP：5’-TCAAATCTGGGCAACTGATGAAAGC-3‘ |
| Sme2.5_01402.1_g00012.1 | LP：5’-TACACCAAAGCAACAGTCAAGG-3‘ |
|  | RP：5’-GGAGAGACATCAAGAGCCATC-3‘ |
| Sme2.5_02075.1_g00005.1 | LP：5’-CGTGAAGGTGGATCAAATCAAAG-3‘ |
|  | RP：5’-CTTTAGATGTTTTTGGAGTTGCTTC-3‘ |
| Sme2.5_05386.1_g00002.1 | LP：5’-AAGAAGGTTCAGATGATGAGTTTTG-3‘ |
|  | RP：5’-ATCTTTCATTTCAGCCTTAATCTCA-3‘ |
| Sme2.5_10866.1_g00001.1 | LP：5’-AACGATCAATGGCACCGTATG-3‘ |
|  | RP：5’-CACAGTAAGCACTCCATTGTCCAT-3‘ |
| Sme2.5_00524.1_g00007.1 | LP：5’-ATTGATTCACTGAAAGCTAAGCTTG-3‘ |
|  | RP：5’-GAGTCATCTTTTCCTTCAATAGCG-3‘ |
| Sme2.5_00678.1_g00014.1 | LP：5’-AACTCAGGCATTGATGCCATAC-3‘ |
|  | RP：5’-GGGATAGTCAGTGGGGTTTGTT-3‘ |
| Sme2.5_01445.1_g00014.1 | LP：5’-GGAGGAATTACAGCTCTTACGACAC-3‘ |
|  | RP：5’-TCTCAACTTTGGTCTTAGGAATCG-3‘ |
| BGI_novel_G012131 | LP：5’-AGAAGTTTATCATTATAGCTTGGTT-3‘ |
|  | RP：5’-GAAGAAGGTAAGAAAACTATACTTC-3‘ |
| Sme2.5_05449.1_g00002.1 | LP：5’-CAAGCATACACTGTGGTATCTGACG-3‘ |
|  | RP：5’-CACTGCATTGAGATGTACCGATGT-3‘ |
| Sme2.5_08297.1_g00002.1 | LP：5’-GCTGGACACCAAGACTCCATTC-3‘ |
|  | RP：5’-TCTCGTTCACTACTTTCATGGGTCT-3‘ |
| Sme2.5_01772.1_g00003.1 | LP：5’-TACCCACAAGACGAATGAGGC-3‘ |
|  | RP：5’-CCATATGCAGGGGTGTTAATTG-3‘ |
| BGI_novel_G020285 | LP：5’-TGTTGGTGAATGGATAAAGAAGAAT-3‘ |
|  | RP：5’-AAGTCCAAACTATAGTTTCAAGGGG-3‘ |
| Sme2.5_12868.1_g00001.1 | LP：5’-CAGAACCAAACACCCTACTACCC-3‘ |
|  | RP：5’-GAATGGACCATCCCTGAAAACT-3‘ |
| Sme2.5_04829.1_g00004.1 | LP：5’-AATGAGGCTTACGGAAGTTGTC-3‘ |
|  | RP：5’-CATAGTCGTAGCCGTGAAAGTTAT-3‘ |
| BGI_novel_G006139 | LP：5’-TCTCCATTTGTAGCAGCACCG-3‘ |
|  | RP：5’-CTTGTCGAGCCCAGGCATAT-3‘ |
| BGI_novel_G007853 | LP：5’-ATGTATCAGCCTTCGTTCTGCAT-3‘ |
|  | RP：5’-AATAAGGGAAACAGCCAACGCC-3‘ |
| BGI_novel_G007427 | LP：5’-GTGGACGGACTTTTACAGATTAC-3‘ |
|  | RP：5’-ATGTTGTACAGTAACTTCTGATGC-3‘ |
| BGI_novel_G004713 | LP：5’-TGGTAGTGGTAGAGGACGAAG-3‘ |
|  | RP：5’-ATAACATTACGTGCGAGCGAGA-3‘ |
| Sme2.5_01406.1_g00001.1 | LP：5’-CCACACTTCTCATATTGCTGTCA-3‘ |
|  | RP：5’-ACCAGCATCACCATTCTTCAAAA-3‘ |
