# Supplementary material for: Transcriptome profiling and gene expression analyses of eggplant (Solanum melongena L.) under heat stress
Source: PLoS One. 2020 Aug 11;15(8):e0236980. doi: 10.1371/journal.pone.0236980 (PMC7419001; doi:10.1371/journal.pone.0236980)
Supplement: S2 Table — (DOC) [file pone.0236980.s006.doc]

**S2 Table**. The prediction of new transcripts

| **Total novel transcript** | **Coding transcript** | **Noncoding transcript** | **Novel isoform** | **Novel gene** |
| --- | --- | --- | --- | --- |
| 25482 | 20641 | 4841 | 0 | 20641 |
